# MEG language mapping using a novel automatic ECD algorithm in comparison with MNE, dSPM, and DICS beamformer

**DOI:** 10.3389/fnins.2023.1151885

**Published:** 2023-06-02

**Authors:** Abbas Babajani-Feremi, Haatef Pourmotabbed, William A. Schraegle, Clifford S. Calley, Dave F. Clarke, Andrew C. Papanicolaou

**Affiliations:** ^1^Department of Neurology, University of Florida, Gainesville, FL, United States; ^2^Magnetoencephalography (MEG) Lab, The Norman Fixel Institute of Neurological Diseases, University of Florida Health, Gainesville, FL, United States; ^3^Department of Neurology, Dell Medical School, University of Texas at Austin, Austin, TX, United States; ^4^Comprehensive Pediatric Epilepsy Center, Dell Children’s Medical Center, Austin, TX, United States; ^5^Department of Pediatrics, Dell Medical School, University of Texas at Austin, Austin, TX, United States; ^6^Department of Neurosurgery, Dell Medical School, University of Texas at Austin, Austin, TX, United States; ^7^Department of Pediatrics, University of Tennessee Health Science Center, Memphis, TN, United States

**Keywords:** magnetoencephalography, language lateralization, single equivalent current dipole, minimum norm estimation, dynamic statistical parametric mapping, dynamic imaging of coherent sources beamformer

## Abstract

**Introduction:**

The single equivalent current dipole (sECD) is the standard clinical procedure for presurgical language mapping in epilepsy using magnetoencephalography (MEG). However, the sECD approach has not been widely used in clinical assessments, mainly because it requires subjective judgements in selecting several critical parameters. To address this limitation, we developed an automatic sECD algorithm (AsECDa) for language mapping.

**Methods:**

The localization accuracy of the AsECDa was evaluated using synthetic MEG data. Subsequently, the reliability and efficiency of AsECDa were compared to three other common source localization methods using MEG data recorded during two sessions of a receptive language task in 21 epilepsy patients. These methods include minimum norm estimation (MNE), dynamic statistical parametric mapping (dSPM), and dynamic imaging of coherent sources (DICS) beamformer.

**Results:**

For the synthetic single dipole MEG data with a typical signal-to-noise ratio, the average localization error of AsECDa was less than 2 mm for simulated superficial and deep dipoles. For the patient data, AsECDa showed better test-retest reliability (TRR) of the language laterality index (LI) than MNE, dSPM, and DICS beamformer. Specifically, the LI calculated with AsECDa revealed excellent TRR between the two MEG sessions across all patients (Cor = 0.80), while the LI for MNE, dSPM, DICS-event-related desynchronization (ERD) in the alpha band, and DICS-ERD in the low beta band ranged lower (Cor = 0.71, 0.64, 0.54, and 0.48, respectively). Furthermore, AsECDa identified 38% of patients with atypical language lateralization (i.e., right lateralization or bilateral), compared to 73%, 68%, 55%, and 50% identified by DICS-ERD in the low beta band, DICS-ERD in the alpha band, MNE, and dSPM, respectively. Compared to other methods, AsECDa’s results were more consistent with previous studies that reported atypical language lateralization in 20-30% of epilepsy patients.

**Discussion:**

Our study suggests that AsECDa is a promising approach for presurgical language mapping, and its fully automated nature makes it easy to implement and reliable for clinical evaluations.

## 1. Introduction

In the last few decades, magnetoencephalography (MEG) has been established as a non-invasive modality for presurgical identification of the irritative zone in patients with epilepsy (Minassian et al., 1999). Additionally, MEG has been used for presurgical mapping of the auditory, visual, motor, and somatosensory cortex and of language-related cortex for assessment of the laterality and localization of language networks (Bowyer et al., 2020a). Multiple mathematical modeling approaches have been developed for source localization using MEG. However, the single equivalent current dipole (sECD) is the most common approach in clinical applications. Other approaches have also been considered and include distributed source models such as minimum norm estimation (MNE; Dikker and Pylkkanen, 2013), dynamic statistical parametric mapping (dSPM; Tanaka et al., 2013), and MR-FOCUSS (Bowyer et al., 2005) as well as beamforming methods such as dynamic imaging of coherent sources (DICS; Foley et al., 2019), linearly constrained minimum variance (LCMV) filtering (Sharma et al., 2021), and synthetic aperture magnetometry (SAM; Hirata et al., 2010).

The sECD approach has been validated for language lateralization against the Wada test (Papanicolaou et al., 2004; Merrifield et al., 2007; Doss et al., 2009; Ota et al., 2011) and for language localization against intraoperative cortical stimulation mapping (Simos et al., 1999). In addition, the sECD approach has been shown to have good inter-rater and test–retest reliability for mapping of receptive language (Breier et al., 2000; Lee et al., 2006). Although the utility and efficiency of the sECD approach for language mapping have been shown in previous studies, this approach has not been widely used in clinical evaluations or in research investigations. A reason for this is that sECD mapping requires subjective judgments in selecting several critical parameters, which may be inconsistent depending on the clinical experience of the investigators (Papanicolaou et al., 2006). In particular, an essential step is the selection of an optimal subset of MEG sensors (separately for the left and right hemispheres) at each of several successive time points in order to fit dipoles based on the measured magnetic fields. To address the technical limitations of the manual sECD approach, we developed an automatic sECD algorithm (AsECDa) that is able to automatically select an optimal subset of sensors at a given time point and determine the required number of dipoles in each hemisphere that adequately models the measured magnetic fields.

We first evaluated the accuracy of the developed AsECDa using synthetic MEG data. Then we compared the performance of AsECDa, MNE, dSPM, and DICS beamformer for language mapping using MEG data recorded during a receptive language task in patients with epilepsy. We assessed the performance of the language mapping methods by evaluating the test–retest reliability of their language laterality estimates and by comparing these estimates against the expected language representation in epilepsy patients determined based on previous studies.

This study, to the best of our knowledge, is the first to compare the performance of the AsECDa for language mapping with that of three other common MEG source modeling techniques (i.e., MNE, dSPM, and DICS beamformer) using the same, clinically acquired, data from a series of epilepsy patients (including children) for whom the recording of good data is challenging. In addition, the inverse solutions of all four source modeling techniques were implemented using the same neuroimaging software [i.e., the FieldTrip toolbox for MATLAB (Oostenveld et al., 2011)] in order to prevent any bias favoring any one approach that might occur with the use of multiple software packages.

## 2. Methods

### 2.1. Patients

Approximately 40 patients with epilepsy and/or brain tumors underwent MEG data collection between June 2021 to March 2022 as part of the presurgical evaluation process at Dell Children’s Medical Center (DCMC), Austin, TX, USA. From these patients, 21 (11 male; 23.0 ± 16.6 [mean ± standard deviation (SD)] years of age; 18 right-handed and three left-handed) were retrospectively selected for inclusion in this study (Table 1). Those included were patients: (a) who underwent MEG presurgical receptive language mapping, (b) who underwent MEG data collection without sedation or general anesthesia, and (c) whose MEG data were not contaminated with artifacts generated by vagus nerve stimulation implantation, orthodontic devices, ventriculoperitoneal shunts, and/or environmental noise. The study was approved by the Institutional Review Board (IRB) of the University of Texas at Austin and Ascension Site Approval Committee to access archival data.

**Table 1:** Demographic and clinical data.

Number of patients (*n*)	21
Male (*n*, %)	11 (52%)
Age (year, mean ± SD)	23.0 ± 16.6
Handedness (*n*, Left/Right)	3/18
Full-scale intelligence quotient [FSIQ] (mean ± SD)	87.3 ± 16.5
Seizure lateralization (*n*, Left/Bilateral/Right)	8/6/7
Seizure/interictal discharge localization in temporal lobe (*n*)	14

### 2.2. Language task protocol

As part of presurgical evaluations in our center, patients completed an auditory word recognition task (WRT) for receptive language mapping. This receptive language task was adapted from the continuous auditory word recognition protocol previously described in Papanicolaou et al. (2004). This task has been routinely used in many clinical centers for MEG language mapping (Pirmoradi et al., 2010). Immediately prior to the beginning of the MEG scan, patients were instructed to listen to and memorize five target words (“*little*,” “*please*,” “*drink*,” “*jump*,” and “*good”*). The target words were presented multiple times until the instructor was reasonably confident that the patients were able to recall the words and follow the instructions. During the MEG recordings, the patients were instructed to close their eyes and lift their right index finger when they recognized one of the five target words. MEG recordings during the same task were repeated in all patients over two consecutive back-to-back sessions (Session 1 and Session 2), which means that the second session started immediately after the first session ended.

The stimuli comprised three blocks, each of which included 40 (non-repeating) distractor words and the five target words in a random order (Supplementary Figure S1). Approximately 60 and 40% of these 45 words were monosyllabic and disyllabic, respectively. The duration of the words was 587 ± 86 [mean ± SD] ms. Stimuli were delivered binaurally via plastic tubes terminating in ear inserts at the patient’s ears and were presented with a randomly varied interstimulus interval (ISI) ranging between 2,000 to 3,000 ms (2,500 ± 280 [mean ± SD] ms).

### 2.3. MEG data acquisition and pre-processing

A whole-head 306-channel MEG system (Elekta Neuromag® TRIUX™, MEGIN Oy [formerly Elekta Oy], Helsinki, Finland) housed in a magnetically shielded room at DCMC was used to collect MEG data. An online high-pass filter of 0.1 Hz and a sampling rate of 1,000 Hz were used. Five head position indicator (HPI) coils were attached to the head and used to determine the head’s position during the data collection. The scalp outline (i.e., headshape) and HPI coil positions were digitized using a 3-D digitizer (Fastrak, Polhemus, Colchester, VT, USA).

MEG data were initially preprocessed using MaxFilter 2.2.12 (Elekta Neuromag® Oy, Helsinki, Finland) to reject environmental noise using the temporal extension of signal space separation (tSSS) method (Taulu and Simola, 2006) with a 10 s sliding window and a correlation threshold of 0.98. The MEG data were then 0.1–170 Hz bandpass filtered, 60 and 120 Hz bandstop filtered, epoched from −1 to 2 s relative to the onset of stimuli, and baseline corrected using a time window from −100 to 0 ms. Trials containing artifacts were removed via visual inspection according to temporal variance, z-score, and kurtosis outliers using a semi-automated artifact identification procedure in the FieldTrip toolbox.^1^ Since the AsECDa is based on the local maxima of the gradients of the magnetic fields, the MEG data were restricted to the 204 gradiometers.

For the AsECDa, MNE, and dSPM, the epoched data were 20 Hz lowpass filtered in order to generate 0.1–20 Hz bandpass filtered data. For the DICS beamformer, we used the 0.1–170 Hz bandpass filtered epochs, which were analyzed in five canonical frequency bands (i.e., alpha [8–12 Hz], low beta [12–20 Hz], high beta [20–30 Hz], low gamma [30–50 Hz], and high gamma [50–110 Hz]). It is noteworthy that in order to compare the efficacy of the different methods (i.e., AsECDa, MNE, dSPM, and DICS) in assessing laterality we were obliged to use each method in the standard way that most experts implement. Specifically, we used a 0.1–170 Hz bandpass filter for the DICS beamformer and then calculated the source power in the five frequency bands using the broadband (i.e., 0.1–170 Hz) MEG signals. While we used 0.1–20 Hz bandpass filter for the other three methods (i.e., ECD, MNE, and dSPM), it is important to note that using a 0.1–170 Hz bandpass filter will not significantly change the time course of the average EMFs in the MEG sensors (see Supplementary Figure S2) and the resultant source localization by these methods. It is also important to note that the concept behind source modeling based on the DICS beamformer is quite different than that for the other three methods (i.e., ECD, MNE, and dSPM); thus, using the exact filtering for them is not a common recommendation for the analysis of MEG data. For example, a low pass filter (e.g., at 20 Hz) will reduce the high frequency noise and artifacts in the EMF signals and has a positive effect on the source localization by the ECD, MNE, and dSPM. However, a low pass filter at 20 Hz cannot be used for the DICS beamformer if we wish to measure the power of brain signals in high beta (20–30 Hz) and gamma (>30 Hz) bands.

**Figure 1:**
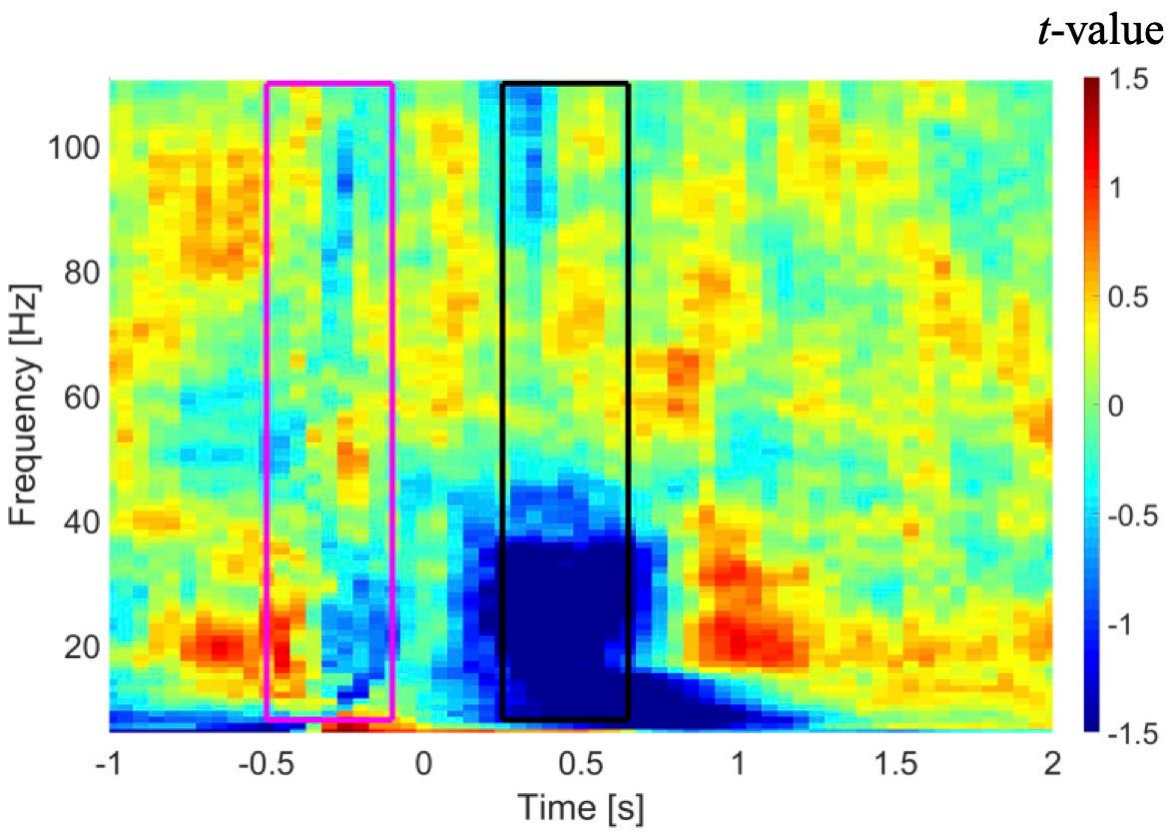
Time-frequency analysis in a representative patient averaged across all gradiometer sensors in the left temporal region. Time zero represents onset of the auditory stimuli. A time window from −500 to −100 ms (purple rectangle) and a time window from 250 to 650 ms (black rectangle) were used as baseline and active intervals, respectively, in DICS beamformer. DICS, dynamic imaging of coherent sources.

Sensor-level time-frequency analysis was conducted to confirm the presence of event-related desynchronization (ERD) and event-related synchronization (ERS), which reflect band-limited power alteration in neuronal oscillations (Ressel et al., 2008; Kadis et al., 2011; Yu et al., 2014; Youssofzadeh and Babajani-Feremi, 2019). Similar to the source-level analyses, only artifact-free epochs were used in the sensor-level time-frequency analysis. A multitaper time-frequency analysis was performed on recordings from all MEG sensors in each patient using the following parameters: (a) taper: discrete prolate spheroidal sequences (DPSS); (b) frequency of interest (FOI): 8 to 110 Hz with steps of 1 Hz; (c) spectral smoothing through multi-tapering: 0.4*FOI (if FOI < 30 Hz) or 12 (if FOI ≥ 30 Hz); (d) length of time window: 7/FOI (if FOI < 30 Hz) or 250 ms (if FOI ≥ 30 Hz); and (e) times of interest: −1 to 2 s. The resulting time-frequency power spectra were baseline-corrected using a −500 to −100 ms time window relative to stimulus onset. The results of the time-frequency analysis in a representative patient are shown in Figure 1. As shown in this figure, a time window from 250 to 650 ms shows suppression of power in lower frequencies, specifically in the alpha and beta bands. Our results revealed an overall suppression of power in alpha and beta bands across subjects, although this suppression had a low consistency across either the two MEG sessions or across the subjects. A trend toward augmentation of power in the gamma band was observed, although this trend was inconsistent across subjects.

### 2.4. MEG forward model

High-resolution T1-weighted anatomical MRI images were co-registered to the MEG data using the surface-matching method in the FieldTrip toolbox. The scalp outline and the three anatomical landmarks (the nasion and the two pre-auricular points) were used to guide co-registration of the MEG data with the MRI data. The co-registered MRI was segmented, and the brain surface from the segmented MRI was used to compute a single-sphere volume conductor model for calculation of the lead-fields for the sECD, DICS beamformer, MNE, and dSPM methods.

For the sECD and DICS beamformer, the source model was defined on a regular 3-D template grid (8,740 points with 4 mm resolution) in normalized Montreal Neurological Institute (MNI) space. The template grid was then volumetrically warped to the native space of the individual using a non-linear transformation based on the spatial normalization of the subject’s T1 image to the standard MNI space. The location of the dipoles for the sECD and the activity of the sources for the DICS beamformer in the template grid were mapped to the Brainnetome volumetric atlas (Fan et al., 2016) to find sources in the language-specific regions-of-interest (ROIs).

A cortical surface was used for the source model for the MNE and dSPM methods. The individual anatomical MRI of each subject was used to generate a cortical surface using the standard FreeSurfer *recon-all* pipeline (Fischl, 2012). In brief, this pipeline performs intensity normalization, skull stripping, subcortical volume generation, gray/white matter segmentation, and parcellation. After running the *recon-all* pipeline, the generated file describing the white-grey matter boundary surface was used in the MNE Suite software package (Matti Hämäläinen, Martinos Center for Biomedical Imaging, Massachusetts General Hospital, MA) to create a set of points that covers the cortical surface based on the topology of a recursively subdivided octahedron by a factor of 6 (“oct-6”). The generated cortical surface had 8,196 points. We used the Destrieux standard atlas (Destrieux et al., 2010), provided by FreeSurfer, to parcellate the MNE and dSPM solutions and find those within the language-specific ROIs.

### 2.5. Automatic sECD algorithm for language mapping

**Figure 2:**
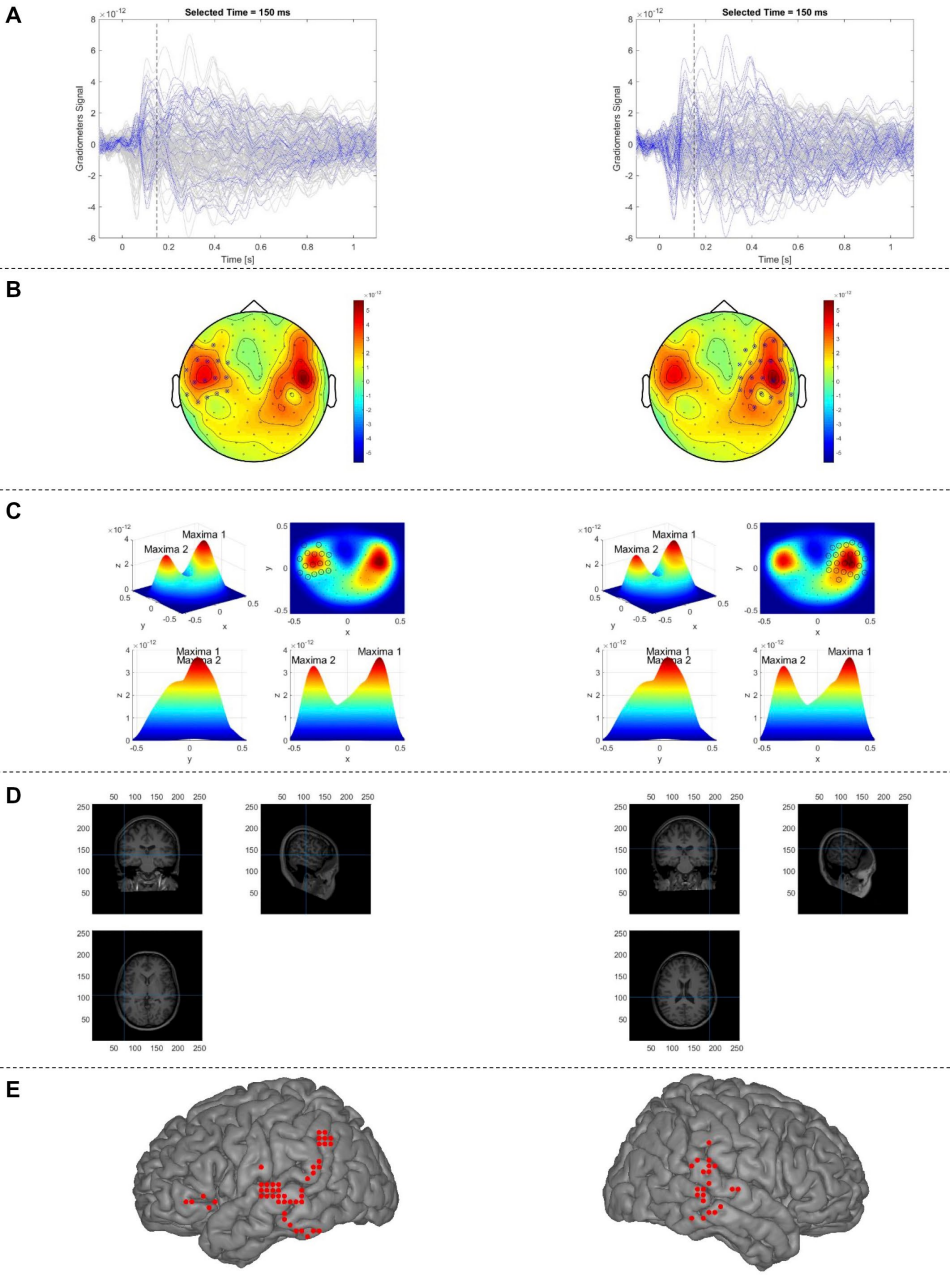
Proposed automatic sECD algorithm (AsECDa) for language mapping using MEG. **(A)** Time courses of the MEG gradiometers; the time courses of the gradiometers selected in **(B)** are in blue color and that of the rest of sensors are in gray color. (b) The 2D topo-plot of MEG gradiometers at a given time point (*t* = 150 ms). The selected optimal subset of MEG sensors (separately for the left and right hemispheres) at the given time point are marked by crossing circles. **(C)** The measured magnetic fields shown in (b) are spatially smoothed using a 2-D Gaussian smoothing kernel, which resulted in two maxima (one in the left and another in the right). The selected optimal subset of MEG sensors is marked by circles. **(D)** Locations of two dipoles (separately for the left and right hemispheres) fitted to the measured magnetic fields of the selected sensors are shown in the native space. **(E)** Locations of all fitted dipoles (in a representative patient) within a 150 to 650 ms time window are shown in standard MNI space. Only language-related dipoles are shown. MEG, magnetoencephalography; MNI, Montreal Neurological Institute; sECD, single equivalent current dipole.

Overall procedures of the automatic sECD algorithm (AsECDa) are shown in Figure 2. The cleaned and averaged evoked magnetic field (EMF) data were submitted to an automatic algorithm to identify at each point in time (every 1 ms) the presence of one or more dipolar magnetic field distributions. At each time point, a group of neighboring MEG channels that best cover each dipolar magnetic field distribution were selected and used to estimate the location, direction, and moment of a dipole source. The automatic channel group selection at each point in time was the core of the proposed automatic algorithm. To select an appropriate group of neighboring channels at a given latency, the algorithm used the magnetic fields of the planar gradiometer channels, leveraging the fact that the dipole sources are generally located below the maxima of the planar gradients of the field. In the MEGIN/Elekta MEG system, there are two perpendicular (i.e., horizontal and vertical) gradiometer channels at each sensor location. The magnetic fields of the two channels were combined to generate a single positive value per sensor location. The 3-D positions of the planar gradiometer channels were projected to a 2-D layout. The measured magnetic fields of these channels were then spatially smoothed using a 2-D Gaussian smoothing kernel with a standard deviation of SD = min(*d*) where *d* is the 2-D distances between all channel pairs. At a given latency, the local maxima and global maximum of the smoothed field pattern were calculated. Up to 5 of the strongest local maxima were retained if their values were at least 10% of the value of the global maximum in order to prevent fitting spurious dipoles with low signal-to-noise ratio.

The selected, up to 5, local maxima were divided into left and right hemisphere subsets, and global maxima for the right and left subsets were calculated separately. For each hemisphere, only maxima with a value of at least 75% of the value of the global maximum in the corresponding hemisphere were retained. For each of the surviving maxima in the left and right hemispheres at a given latency, neighboring channels were selected and used to fit a single dipole. To select the neighboring channels around the central channel at a local maximum, a 1-D Gaussian curve was fit to the measured magnetic fields as a function of the 2-D distances between the central channel and the other channels. Only channels with a normalized distance of no more than 0.4 were included in the 1-D Gaussian fit. A minimum and maximum cutoff for the radius around the central local maximum channel (*Rad_Cutoff*) was calculated as follows:

(1) $$Rad\_Cutoff=\min\;\left(\max\;\left(\begin{array}{l} Sigma\_Factor\cdot \sigma ,\\ \min\_Rad \end{array}\right),\max\_Rad\right)$$

where *σ* is the standard deviation of the estimated 1-D Gaussian curve and the parameters *Sigma_Factor*, *Min_Rad*, and *Max_Rad* were set to 1.0, 0.1, 0.25, respectively. All channels with a distance less than *Rad_Cutoff* were selected and used for dipole fitting as implemented using the “*ft_dipolefitting*” function of the FieldTrip toolbox. The whole brain is initially scanned with a single dipole on the individual grid (8,740 points at a 4 mm resolution) to find the optimal starting location, and a non-linear search algorithm is then used to find the location and moment of the dipole that best explains the measured magnetic field pattern in the selected channel group.

The dipoles corresponding to the local maxima of the field pattern were fitted at each successive time point (every 1 ms). Only dipoles that met our criteria of acceptability (correlation between the estimated and measured MEG fields in the selected channels ≥0.90; and relative residual variance of the fitted dipole <20%) were kept and submitted to a spatiotemporal smoothing process, motivated by Papanicolaou et al. (2004), to promote spatiotemporally connected dipoles and remove isolated dipoles. As part of this process, the following rank measure was calculated:

(2) $$\{\begin{array}{c} Rank_{i}=\overset{n}{\underset{j,j\neq i}{\sum }}Gs_{ij}\cdot Gt_{ij}\\ Gs_{ij}=e^{-\left(\frac{d_{ij}^{2}}{2\sigma_{s}^{2}}\right)},Gt_{ij}=e^{-\left(\frac{t_{ij}^{2}}{2\sigma_{t}^{2}}\right)} \end{array}$$

where *Rank_i_* is the ranking factor for the *i*th dipole, *n* is the total number of dipoles in the time window of interest, *d_ij_* and *t_ij_* are the 3-D distance and time difference, respectively, between the *i*th and *j*th dipoles, and *σ_s_ = 10 mm* and *σ_t_ = 50 ms* are standard deviations of the Gaussian kernel for spatial and temporal smoothing. Only dipoles within the top 70% ranking factor were retained for computation of the laterality index.

To determine whether the dipoles are within the language-specific ROIs, the locations of the dipoles were transformed into MNI standard coordinate space and then parcellated using the 246 (210 cortical, 36 subcortical) ROIs of the Brainnetome atlas. 50 ROIs in each hemisphere were selected as the language-specific ROIs (Supplementary Table S1a). For a specific time-window of interest (e.g., 150 to 600 ms in this study), the laterality index (LI) was calculated as follows:

(3) $$LI=\frac{L-R}{L+R}$$

where *L* and *R* are the number of dipoles in the left and right language-specific ROIs, respectively.

### 2.6. MNE and dSPM for language mapping

The MNE (Hamalainen and Ilmoniemi, 1994) and dSPM (Dale et al., 2000) algorithms were used to reconstruct source activities from the cleaned and averaged EMF data. The MNE was calculated by applying a linear inverse operator *W* to the measured EMF signals:

(4) $$\hat{s}\left(t\right)=Wx\left(t\right)$$

where *x*(*t*) represents the measured MEG data at time *t* and *s*(*t*) is the corresponding current values in the cortical surface source space. The inverse operator ***W*** in the MNE solution can be obtained as follows:

(5) $$W=RA^{T}{\left(ARA^{T}+\lambda^{2}C\right)}^{-1}$$

where ***A*** is the lead field matrix (calculated based on a cortical surface with 8,196 source locations as described in Section 2.4), ***C*** and ***R*** are covariance matrices of the noise and source activities, respectively, and *λ^2^* is a regularization parameter (Liu et al., 2002). Minimum norm estimates of source activity were obtained based on the “*minimumnormestimate”* function in the FieldTrip toolbox. No orientation constraints were applied to the source. An identity matrix was assigned to the source covariance matrix ***R*** since there was no *a priori* assumption (e.g., from fMRI) on the spatial distribution of the source currents (Dale et al., 2000). The source covariance matrix ***R*** was than scaled such that $\frac{trace\left(ARA^{T}\right)}{trace\left(C\right)}=1$ . The noise covariance matrix ***C*** was estimated from the empty room noise data, which were collected on the same day before scanning the patients. The lead field matrix ***A*** was pre-whitened with the noise covariance matrix ***C***. The regularization parameter *λ^2^* was set to the suggested default value in the FieldTrip toolbox (i.e., *λ^2^* = 3).

The calculated inverse operator ***W*** from the MNE solution was then used to compute the noise-normalized current density estimate, which is the dSPM solution (Dale et al., 2000):

(6) $$q_{i}\left(t\right)=\frac{\sum_{j\epsilon G_{i}}{\left(w_{j}\;x\left(t\right)\right)}^{2}}{\sum_{j\epsilon G_{i}\;}w_{j}Cw_{j}^{T}}$$

where *q_i_*(*t*) is the noise-normalized estimate of the local current dipole power (sum of squared dipole component strengths) at the *i*^th^ location, *G_i_* is the set of three dipole component indices for the *i*^th^ location, and *w_j_* represents the row of the inverse operator *W* corresponding to the *i*^th^ location and *j*^th^ dipole component. *Q_i_*(*t*) has a F-distribution under the null hypothesis, with the numerator having three degrees of freedom and the denominator having degrees of freedom that are typically very large, depending on the number of time samples used for calculation of the noise covariance matrix *C* (Dale et al., 2000).

The power of the MNE and dSPM source activity (averaged within the time window of interest, which was 150 to 600 ms in this study) was used to calculate the laterality index (LI) based on the Destrieux standard atlas (Destrieux et al., 2010). The Destrieux atlas divides the cerebral cortex into 75 parcels per hemisphere, giving a total of 150 parcels. 25 ROIs in each hemisphere were selected as the language-specific ROIs (Supplementary Table S1b). The LI for MNE and dSPM was calculated based on Eq. (3) where L and R were the average power of source activity in the left and right language-specific ROIs, respectively. Considering that a null distribution (i.e., F-distribution) is known for the dSPM solution, the source activity resulting from this method was first thresholded using a *value of p* of 0.05 (Bonferroni corrected for multiple comparisons in 8196 source locations) before computation of the LI.

### 2.7. Language mapping using DICS beamformer

The DICS method, an adaptive spatial filtering (i.e., beamforming) technique in the frequency-domain, was initially introduced as a variant of the LCMV beamformer to facilitate analysis of oscillatory source activity and the connectivity between sources via coherence (Gross et al., 2001). Although the LCMV method with properly filtered data should provide results similar to that of the DICS, the DICS beamformer is preferable when narrow band-limited power of source activity is investigated (Westner et al., 2022). In the current study, the DICS beamformer was used to localize and lateralize language based on band-limited power changes in neuronal oscillations in five canonical frequency bands (i.e., alpha [8–12 Hz], low beta [12–20 Hz], high beta [20–30 Hz], low gamma [30–50 Hz], and high gamma [50–110 Hz]).

A vector version of the DICS beamformer was used in this study. The estimated source activity $\hat{S}\left(r,f\right)$ at location 𝑟 and frequency of interest 𝑓 can be computed as follows:

(7) $$\hat{S}\left(r,f\right)=W{\left(r,f\right)}^{T}X\left(f\right)$$

where ***X***(*f*) denotes the frequency domain sensor signals at a given frequency 𝑓 and the *n*-by-3 matrix ***W***(*r*,*f*) is the DICS spatial filter at location 𝑟 and frequency 𝑓, with three columns referring to the 𝑥, 𝑦, and 𝑧 components of the dipole moment and *n* to the number of MEG channels. The spatial filter weight matrix ***W***(*r*,*f*) can be computed as follows:

(8) $$W^{T}\left(r,f\right)={\left[A^{T}\left(r\right)Q{\left(f\right)}^{-1}A\left(r\right)\right]}^{-1}A^{T}\left(r\right)Q{\left(f\right)}^{-1}$$

where the *n*-by-*n* matrix ***Q***(*f*) describes the cross-spectral density (CSD) of the MEG sensors for frequency 𝑓 and ***A***(*r*) denotes the *n*-by-3 forward lead field matrix at location 𝑟 for the three directions 𝑥, 𝑦, and 𝑧 (Gross et al., 2001). The complex-valued CSD matrix describes the shared power and phase shift between the signals of the different sensors at a given frequency 𝑓 and can be calculated as follows:

(9) $$Q\left(f\right)=X\left(f\right).X^{H}\left(f\right)$$

where $X^{H}$ refers to the Hermitian (i.e., complex conjugate) transpose of matrix ***X***. As recommended in previous studies such as Westner et al. (2022), we only considered the real-valued part of the CSD matrix ***Q***(*f*) in Eq. (8) to prevent having a complex-valued spatial filter, which does not have a valid biophysical interpretation.

The cleaned broad-band (bandpass filtered at 0.1–170 Hz) epochs of MEG sensor signals were used in the DICS beamformer to calculate the alteration of power in the five frequency bands from pre-stimulus (i.e., baseline) to post-stimulus (i.e., active) conditions. We selected a time window from −500 to −100 ms for pre-stimulus and from 250 ms to 650 ms for the post-stimulus based on the results of time-frequency analysis in the sensor space (see Figure 1). To compare the two conditions statistically, the sources were estimated based on an inverse filter [i.e., weight ***W***(*r*,*f*)*^T^*] in Eq. (7) that was computed from combining the two conditions (called the common filter approach), and then the common filter was applied separately to each condition to estimate the source power. The CSD matrix ***Q***(*f*) for the baseline, active, and combined conditions were calculated using the fast Fourier transform (FFT) and a conventional single taper (i.e., Hanning) approach. The regularization parameter for calculating ***Q***(*f*)^−1^ in Eq. (8) was set to the suggested default value in the FieldTrip [i.e., 10% of the average of the eigenvalues of ***Q***(*f*)]. We conducted a DICS beamformer analysis for lambda values ranging from 1 to 15% and found that altering the value did not affect the laterality assignment. Additionally, we tested the DICS beamformer on MEG data without employing the tSSS filter, but observed no improvement in the results (refer to Supplementary Figure S3; Supplementary Table S2 in the Supplementary materials).

To calculate the source power in each condition, the source CSD was calculated as the 3-by-3 matrix ***W***(*r*,*f*)*^T^****Q***(*f*)***W***(*r*,*f*) and then projected along the dominant direction, corresponding to the largest eigenvalue of the matrix (Gross et al., 2001). For each condition, the average of the source power across frequencies in each of the five bands was calculated.

The statistical inference for the source power in each frequency band was performed using a non-parametric randomization test based on a Monte-Carlo estimate of the probability distribution of the change in the source power. The significance of a dependent sample *t*-statistic for the source power was evaluated by randomly permuting (*n* = 10,000) the baseline and active conditions in order to generate an empirical null distribution. Two statistical approaches were used to find significant language sources: (I) an uncorrected *p* < 0.05; and (II) a cluster-based correction for multiple comparisons with an alpha level of 0.05. The significantly active sources in each of the five frequency bands was used to calculate the LI. Similar to the sECD method, 50 ROIs in each hemisphere were selected from the Brainnetome atlas as the language-specific ROIs. The LI was calculated based on Eq. (3) where L and R were the number of statistically significant sources in the left and right language-specific ROIs, respectively.

ERD and ERS are typically investigated for band-limited power modulation in many cognitive tasks, including language (Pfurtscheller, 1997). ERD and ERS reflect decreases and increases in spectral power relative to a baseline, respectively. In the current study, the LI was calculated separately for ERD and ERS in each frequency band as previous studies suggest that the ERD and ERS may reflect different aspects of the language network (Sharma et al., 2021).

### 2.8. Simulation for sECD

The performance of the MNE, dSPM, and DICS for MEG source localization has been investigated in several studies using simulated MEG datasets (Gross et al., 2001; Lin et al., 2006; Schoffelen and Gross, 2009; Hauk et al., 2011; Hincapie et al., 2017). In this study, we evaluated the localization accuracy of the proposed AsECDa using synthetic MEG datasets. In particular, we aimed to evaluate the localization accuracy for superficial sources as well as for deep sources (e.g., hippocampus), which are typically considered difficult to localize with MEG.

In the simulated MEG datasets, a single-sphere volume conductor model was used for calculation of the lead-fields. Computation of the lead-fields requires knowledge of the locations and orientations of the MEG sensors and possible sources. For this analysis, a source was simulated as a single dipole located at the centroid of one of the 246 ROIs of the Brainnetome atlas. At each location, the simulation was performed separately for two current dipoles oriented in two orthogonal directions (corresponding to the directions of the two principal components of the lead-field matrix in the dipole’s location). We used the head model of a representative subject (i.e., Subject #1), which was generated in the pre-processing step (Section 2.4), for the locations and orientations of the MEG sensors in the simulated datasets.

The synthetic signals for the MEG sensors were computed for each of the 246 locations (i.e., ROIs) and two dipole orientations. This was repeated multiple times after adding random white noise (*n* = 100 repetitions) with different signal-to-noise ratios (SNRs) from 1 to 10 (in steps of 1) and at infinite SNR (i.e., without noise). The proposed AsECDa was then used to localize a dipole corresponding to the synthetic MEG data. Mean and standard deviation of the localization error across the 100 repetitions of the random noise (at a given SNR) were calculated.

### 2.9. Software implementation

The analysis was implemented using in-house software developed in MATLAB R2018b (MathWorks Inc., Natick, MA, USA) and adapted from the following open-source toolboxes. The FieldTrip toolbox v20180905 (Oostenveld et al., 2011) was used to preprocess the MEG data, co-register MEG and structural MRI, compute the head model and lead-fields, and perform source reconstruction using the sECD, MNE, dSPM, and DICS beamformer methods. For the MNE and dSPM methods, FreeSurfer (version 5.3.0, https://surfer.nmr.mgh.harvard.edu) and MNE Suite (Version 2.7.4, M. Hämäläinen, Martinos Center for Biomedical Imaging, Massachusetts General Hospital, MA) were used to generate the cortical surface for each patient and to find sources within the language-specific ROIs based on the Destrieux standard atlas.

## 3. Results

### 3.1. sECD simulation

**Figure 3:**
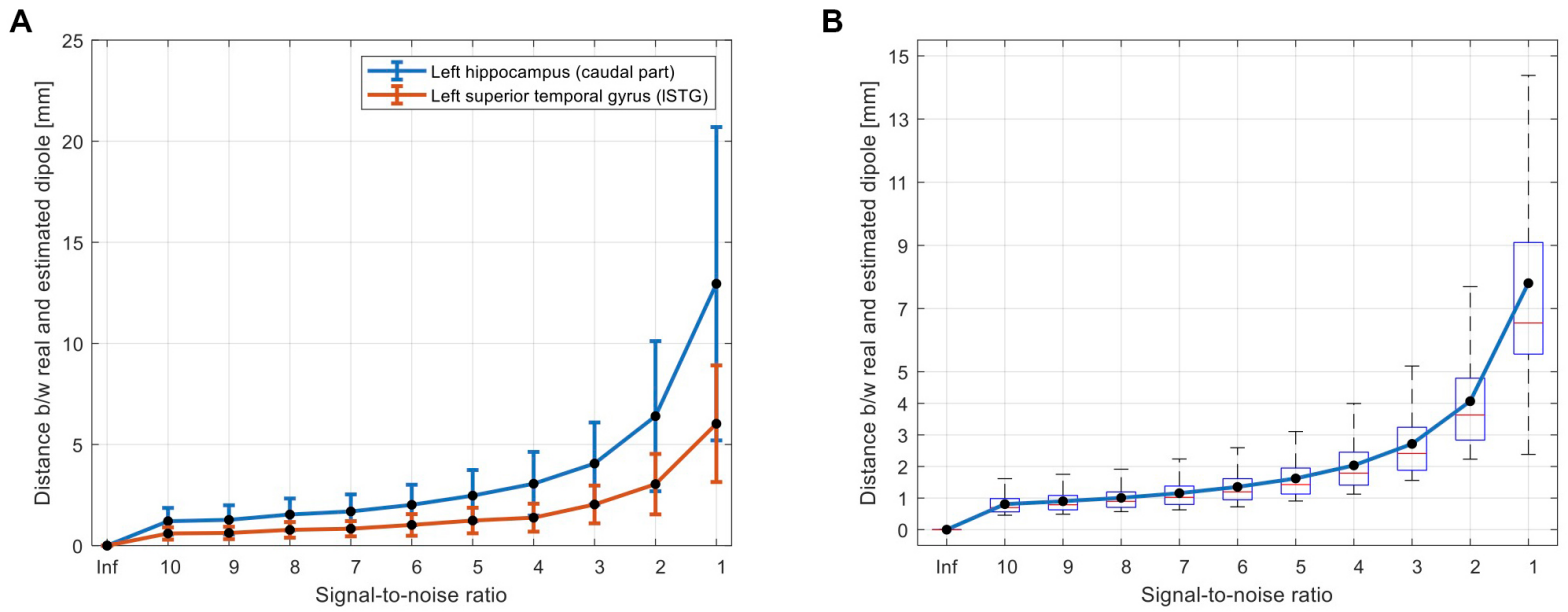
Simulation results for evaluation of accuracy of the proposed AsECDa for localization of a single dipole with different SNRs from 1 to 10 (in steps of 1) and a SNR of infinity (“Inf”; i.e., without noise). **(A)** Dipole localization errors for a deep source (i.e., hippocampus) and a superficial source (i.e., superior temporal gyrus) are compared. The error bars represent standard deviation of the localization error across *n* = 100 repetitions of adding random white noise with a specific SNR. **(B)** Localization errors across all 246 dipoles located in the centroid of 246 ROIs of the Brainnetome atlas. AsECDa, automatic single equivalent current dipole algorithm; ROIs, regions of interest; SNR, signal-to-noise ratio.

Figure 3 shows the performance of the proposed automatic algorithm for localization of simulated dipoles. As expected and shown in Figure 3, the localization error was increased (a) by increasing noise level (and decreasing SNR) and (b) for the deep sources compared to the superficial sources [e.g., hippocampus vs. the superior temporal gyrus (Figure 3A)]. Considering a typical SNR = 5 for EMF data (Lin et al., 2004), the average localization error of the proposed algorithm across all superficial and deep sources (Figure 3B) was less than 2 mm (1.6 ± 0.6). Even in a very noisy condition (i.e., SNR = 1), the proposed algorithm performed well with an average localization error of less than 8 mm (7.8 ± 3.6).

### 3.2. DICS beamformer statistical analyses

The DICS beamformer was used to lateralize and localize language, based on suppression (DICS-ERD) and augmentation (DICS-ERS) of power in the five frequency bands. A summary of the results for the two sessions in all patients using the two statistical approaches (i.e., using an uncorrected *value of p* and a cluster-based correction for multiple comparisons) are listed in Table 2. Using the cluster-based correction with a false alarm rate of 0.05, the results in Table 2B indicate the following: (I) on average, more than 76% of the sessions in all patients across the five frequency bands and two DICS approaches (i.e., DICS-ERD and DICS-ERS) did not result in any significant source within the language cortex; (II) DICS-ERS did not result in any significant language source in the five frequency bands in more than 95% of the sessions; and (III) for DICS-ERS in all frequency bands and DICS-ERD in the high gamma band, only a small portion of the language cortex (< 1.5%) survived after applying statistical thresholding. Referring to these results, the cluster-based statistics for the DICS beamformer did not provide reliable results for language lateralization, and, thus, we did not report results based on this statistical thresholding approach hereafter.

**Table 2:** Average language laterality index (LI) and percentage of active sources in different frequency bands for DICS beamformer using (A) uncorrected *p*-value <0.05 and (B) cluster corrected *p*-value <0.05 statistical thresholding methods.

		LI	Percentage of volume with significantly active sources			Percentage of sessions without significant language source
			Language areas	Non-language areas	All cortical areas	
(A)						
DICS-ERD	Alpha	0.01 ± 0.50	36.4 ± 27.0	25.0 ± 23.4	29.2 ± 23.7	2.4
	Low beta	−0.01 ± 0.48	36.8 ± 28.9	26.8 ± 24.8	30.5 ± 25.6	2.4
	High beta	0.06 ± 0.64	22.6 ± 24.2	22.4 ± 21.0	22.5 ± 21.4	4.8
	Low gamma	0.08 ± 0.84	5.8 ± 8.7	7.7 ± 10.7	7.0 ± 9.6	9.5
	High gamma	0.04 ± 0.90	1.9 ± 2.8	2.5 ± 4.0	2.3 ± 3.3	38.1
DICS-ERS	Alpha	0.06 ± 0.93	0.5 ± 1.3	1.8 ± 4.5	1.3 ± 3.2	59.5
	Low beta	0.07 ± 0.95	0.9 ± 2.2	1.4 ± 2.7	1.2 ± 2.3	50
	High beta	0.12 ± 0.85	3.3 ± 7.7	2.1 ± 6.2	2.5 ± 6.3	38.1
	Low gamma	−0.05 ± 0.85	5.3 ± 9.7	2.3 ± 3.8	3.4 ± 5.8	16.7
	High gamma	−0.04 ± 0.86	3.5 ± 5.3	3.4 ± 5.0	3.4 ± 4.3	11.9
(B)						
DICS-ERD	Alpha	−0.11 ± 0.50	32.2 ± 29.7	23.4 ± 24.5	26.6 ± 25.4	28.6
	Low beta	−0.02 ± 0.47	32.8 ± 31.5	24.3 ± 26.5	27.4 ± 27.8	35.7
	High beta	0.23 ± 0.56	20.4 ± 25.0	20.2 ± 22.1	20.2 ± 22.4	38.1
	Low gamma	0.52 ± 0.39	3.4 ± 8.5	4.0 ± 10.4	3.7 ± 9.5	83.3
	High gamma	0.03 ± 0	0.2 ± 1.6	0.5 ± 3.2	0.4 ± 2.6	97.6
DICS-ERS	Alpha	0.67 ± 0	0.1 ± 0.6	0.4 ± 2.7	0.3 ± 1.9	97.6
	Low beta	-	-	-	-	100
	High beta	0.63 ± 0.52	1.2 ± 5.8	1.0 ± 5.9	1.1 ± 5.8	95.2
	Low gamma	0.10 ± 0.75	1.5 ± 6.6	0.7 ± 3.1	1.0 ± 4.4	95.2
	High gamma	−1.00 ± 0.00	0.6 ± 2.9	0.2 ± 1.1	0.4 ± 1.7	95.2

DICS, dynamic imaging of coherent sources; ERD, event-related desynchronization; ERS, event-related synchronization.

As expected and depicted in Table 2, the statistical analysis based on an uncorrected *p*-value (Table 2A) resulted in more significant DICS sources compared to that obtained when using the cluster-based correction (Table 2B). It is notable that some of the significant sources identified by the uncorrected *p*-value approach could be false positives. The results based on an uncorrected *p* < 0.05 in Table 2A indicate the following: (I) more than 38% of the sessions did not result in any significant language source for DICS-ERD in the high gamma band and for DICS-ERS in the alpha, low beta, and high beta bands; and (II) DICS-ERD in the high gamma band and DICS-ERS in all five frequency bands resulted in significant sources located in a small portion of the language cortex (≤5%) and non-language cortex (≤3%). The small percentage of sources in the language and non-language cortices are comparable to the expected 5% false positives based on the *p* < 0.05. Referring to these observations, the results of DICS-ERD in the high gamma band and DICS-ERS in the five frequency bands should be considered with caution as they may reflect false positive effects.

The results based on an uncorrected *p* < 0.05 in Table 2A revealed that approximately 36 and 37% of the language cortex and 25 and 27% of the non-language cortex, on average across all session, had significant suppression (DICS-ERD) of power in the alpha and low beta bands, respectively. This observation indicates that suppression of power in alpha and low beta bands may be sensitive but not specific for identification of the language cortex.

### 3.3. Language laterality using sECD, MNE, dSPM, and DICS

**Figure 4:**
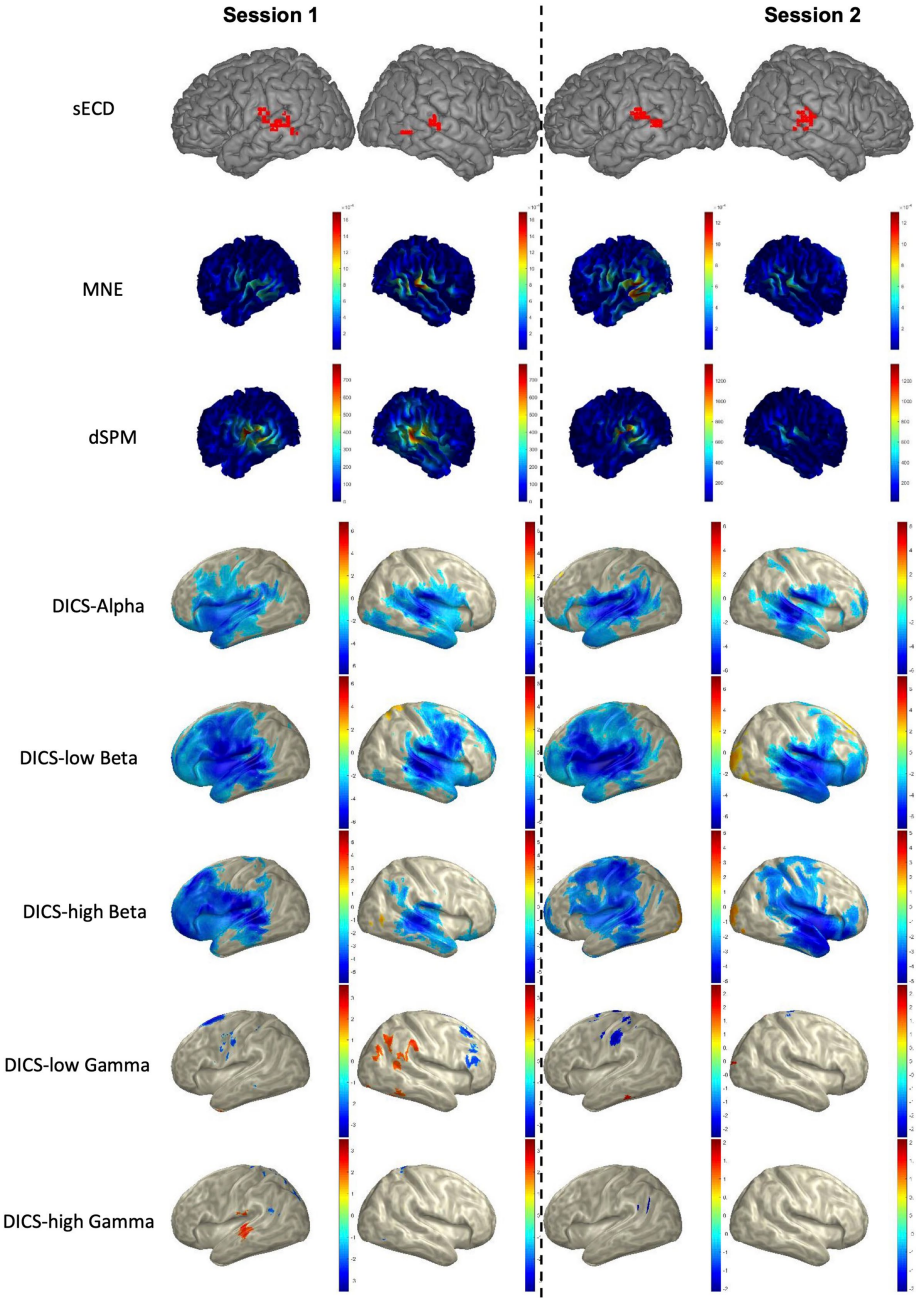
Language mapping using sECD, MNE, dSPM, and DICS beamformer in a representative patient. For AsECDa, locations of all fitted language dipoles within 150 to 650 ms time window are shown in the MNI standard atlas. For MNE and dSPM, the average source activity from 150 to 650 ms is shown in the subject’s native space. For DICS beamformer, the average suppression (blueish color) and enhancement (reddish color) of source activity from baseline (−500 to −100 ms) to the post-stimulus (250 to 650 ms) in five frequency bands are shown in the inflated MNI space. AsECDa, automatic single equivalent current dipole algorithm; dSPM, dynamic statistical parametric mapping; DICS, dynamic imaging of coherent sources; MNE, minimum norm estimation; MNI, Montreal Neurological Institute; sECD, single equivalent current dipole.

The LI was calculated for each of the two sessions in all patients (total of 2×21 = 42 sessions) based on the AsECDa, MNE, dSPM, and DICS beamformer approaches (Table 3; Figure 4). EMFs based on the 0.1–20 Hz bandpass filter were used for AsECDa, MNE, and dSPM. The AsECDa provided a strong and significant correlation between the calculated LIs of Session 1 and Session 2 across all patients (Pearson’s correlation coefficient [Cor] = 0.80; *p* < 0.00002), which shows an excellent test–retest reliability of this approach for determination of language laterality. The intersession correlation of the LI was lower but still good for the MNE (Cor = 0.71; *p* < 0.0004) and dSPM (Cor = 0.64; *p* < 0.002). The LI for the DICS-ERD in the alpha and low beta bands had a significant (*p* < 0.05) but much lower intersession correlation (Cor = 0.64 and 0.48, respectively). These results show a fair to good test–retest reliability for determination of language laterality based on MNE/dSPM and DICS-ERD in the alpha and low beta bands. The intersession correlation of the LI for DICS-ERD in the high beta and gamma bands and for DICS-ERS in all the frequency bands was not significant (*p* > 0.1). In general, compared to the AsECDa and MNE/dSPM methods, the DICS beamformer provided an inferior test–retest reliability for the LI.

**Table 3:** Language laterality results for AsECDa, MNE, dSPM, DICS-ERD, and DICS-ERS.

	AsECDa		MNE		dSPM		DICS-ERD										DICS-ERS									
							Alpha		Low Beta		High beta		Low gamma		High gamma		Alpha		Low beta		High beta		Low gamma		High gamma	
	S1	S2	S1	S2	S1	S2	S1	S2	S1	S2	S1	S2	S1	S2	S1	S2	S1	S2	S1	S2	S1	S2	S1	S2	S1	S2
Number of Sessions w Left Laterality	15	11	9	10	11	10	5	8	5	6	9	8	10	10	8	5	5	4	5	6	9	5	8	7	9	7
Number of Sessions w Right Laterality	4	6	2	3	2	4	6	5	7	5	7	5	7	8	5	6	2	6	5	5	5	6	6	10	8	9
Number of Bilateral Sessions	2	4	10	8	8	7	9	8	9	9	4	7	1	2	1	1	0	0	0	0	0	1	2	2	1	3
Percentage of Sessions w Left Laterality	62%		45%		50%		32%		27%		43%		53%		50%		53%		52%		54%		43%		43%	
Percentage of Sessions w Right Laterality	24%		12%		14%		27%		29%		30%		39%		42%		47%		48%		42%		46%		46%	
Percentage of Bilateral Sessions	14%		43%		36%		41%		44%		28%		8%		8%		0%		0%		4%		11%		11%	
Percentage of sessions w/o Language Source	0%		0%		0%		2%		2%		5%		10%		38%		60%		50%		38%		17%		12%	
Correlation of LI between Sessions 1 and 2	0.80		0.71		0.64		0.54		0.48		0.36		0.19		−0.08		0.00		−0.14		−0.10		0.13		−0.14	
*p*-value of Correlation of LI between Sessions 1 and 2	< 0.00002		< 0.0004		< 0.002		0.015		0.033		0.117		0.458		0.844		0.995		0.734		0.787		0.638		0.615	
LI [Mean ± STD]	0.26 ± 0.58		0.15 ± 0.35		0.16 ± 0.32		0.01 ± 0.50		−0.01 ± 0.48		0.06 ± 0.64		0.08 ± 0.84		0.04 ± 0.90		0.06 ± 0.93		0.07 ± 0.95		0.12 ± 0.85		−0.05 ± 0.85		−0.04 ± 0.86	

AsECDa, automatic single equivalent current dipole algorithm; dSPM, dynamic statistical parametric mapping; DICS, dynamic imaging of coherent sources; ERD, event-related desynchronization; ERS, event-related synchronization; LI, laterality index; S1, session 1; S2, session 2.

In order to investigate the similarity of the results of the different source reconstruction methods, we calculated the correlation between the calculated LIs of all pairs of AsECDa, MNE, dSPM, and DICS approaches in all patients (Table 4). Results in Table 4 showed that the LIs calculated with the MNE and dSPM had a close to perfect correlation (Cor = 0.92; *p* < 10^−16^), which is expected considering the similarity of their inverse solution methods. Our results also revealed a fair to good correlation between the calculated LIs of AsECDa with MNE (Cor = 0.56; *p* < 0.001) and dSPM (Cor = 0.66; *p* < 0.001). We, however, found that there was a poor correlation (Cor < 0.32; *p* > 0.04) between DICS beamformer and the other three approaches (i.e., AsECDa, MNE, and dSPM). The overall similarity of the language laterality calculated using the AsECDa, MNE, and dSPM methods may be in part because the source activities estimated by these approaches are all derived from the EMF signals. The dissimilarity between the EMF-based approaches (i.e., AsECDa, MNE, and dSPM) and the DICS-ERD/ERS beamformer may indicate that the EMFs and ERD/ERS are related to different spatiotemporal neural mechanisms (Pfurtscheller, 1997). In fact, each externally or internally-paced event results in a phase-locked response in the MEG data as an EMF and in a non-phase-locked response in the MEG data as an ERD/ERS.

**Table 4:** Correlation between language laterality index (LI) of all pairs of AsECDa, MNE, dSPM, DICS-ERD, and DICS-ERS methods across both sessions in all patients (i.e., across 42 sessions).

		MNE	dSPM	DICS-ERD					DICS-ERS				
				Alpha	Low beta	High beta	Low gamma	High gamma	Alpha	Low beta	High beta	Low gamma	High gamma
	AsECDa	**0.56** ^**†**^	**0.66** ^**†**^	0.23	0.26	−0.22	0.02	−0.01	−0.03	−0.02	0.04	0.25	0.19
	MNE		**0.92** ^**‡**^	0.32	0.32	−0.06	0.10	−0.18	−0.17	0.16	0.10	0.22	0.28
	dSPM			0.27	0.26	0.08	0.25	−0.18	−0.14	0.21	0.10	0.14	0.21
DICS-ERD	Alpha				**0.56** ^**†**^	−0.18	−0.02	0.00	**−0.74** *	−0.27	0.27	0.27	0.23
	Low beta					0.11	0.15	0.13	−0.06	−0.26	−0.08	0.22	0.01
	High beta						0.54	0.05	0.17	0.00	−0.22	**−0.57** ^**†**^	−0.26
	Low gamma							−0.14	−0.07	−0.03	−0.30	−0.28	−0.15
	High gamma								−0.02	−0.67	−0.03	0.12	−0.11
DICS-ERS	Alpha									0.06	−0.35	0.19	−0.21
	Low beta										0.57	−0.15	−0.09
	High beta											0.26	0.39
	Low gamma												0.23

AsECDa, automatic single equivalent current dipole algorithm; dSPM, dynamic statistical parametric mapping; DICS, dynamic imaging of coherent sources; ERD, event-related desynchronization; ERS, event-related synchronization.

‡ *p* < 10^−16^;

† *p* < 0.001;

* *p* < 0.01. Bold values indicate a significant correlation (*p* < 0.01).

Results in Table 4 show a significant positive correlation between the LIs calculated with the DICS-ERD in the alpha and low beta bands (Cor = 0.56; *p* < 0.001). These findings are in line with the similarity in performance of the DICS-ERD in the alpha and low beta bands regarding the test–retest reliability and the percentage of atypical language representation (Table 3). We also found significant negative correlations between the DICS-ERD and DICS-ERS in the alpha band (Cor = −0.74; *p* < 0.01) and between the DICS-ERD in the high beta and the DICS-ERS in the low gamma band (Cor = −0.57; *p* < 0.001). These significant negative correlations should be considered with caution as results in Table 3 indicate a poor and non-significant test–retest reliability for the LIs calculated with the DICS-ERS in the alpha and low gamma bands.

Based on the LIs calculated using the AsECDa, MNE, dSPM, and DICS beamformer for the two sessions in all patients, the percentage of sessions with left laterality, right laterality, or bilateral representation and the average and standard deviation of the LI across all sessions were computed and reported in Table 3. The DICS-ERD and ERS were biased toward atypical language representation (LI ≈ 0). In particular, the DICS-ERD in the alpha and low beta bands (which provided the most reliable results for the DICS beamformer) were biased toward an atypical language representation as: (a) more sessions were estimated as bilateral language representation rather than left laterality, (b) approximately the same numbers of sessions were estimated as left or right laterality, and (c) approximately two thirds of the sessions were estimated as right laterality or bilateral language representation. These results for DICS are in disagreement with previous studies showing right laterality and bilateral language representation in only 20–30% of epilepsy patients (Gaillard et al., 2007; Moddel et al., 2009).

A mild bias toward atypical language representation was also observed in MNE, for which more sessions were assigned to right laterality or bilateral language representation (≈ 55%) compared to left laterality (≈ 45%). The bias toward atypical language representation was reduced in dSPM, which assigned 50% of the sessions to left laterality. The AsECDa was the least biased toward atypical representation with an LI ≈ 0.26 and 62, 14, and 24% of the sessions assigned to left laterality, bilateral representation, and right laterality, respectively. This more closely agrees with the expected laterality of epilepsy patients as reported in previous studies.

## 4. Discussion

Discrete source models based on sECD have been the primary method used in clinical MEG applications such as language lateralization (Papanicolaou et al., 1999). Distributed solutions are more commonly used in research applications in language and have not yet been fully embraced by the clinical MEG community for language localization and lateralization. MNE (Dikker and Pylkkanen, 2013), dSPM (Tanaka et al., 2013), and MR-FOCUSS (Bowyer et al., 2005) are the main imaging distributed source models and LCMV (Sharma et al., 2021), DICS (Foley et al., 2019), and SAM (Hirata et al., 2010) are the main beamforming methods that have previously been used to investigate language. We selected MNE and dSPM as representative methods for the distributed source models and compared the performance of these methods and the developed AsECDa in the current study for language mapping.

While the AsECDa and other distributed source models, such as MR-FOCUSS and standardized low-resolution brain electromagnetic tomography (sLORETA; Pascual-Marqui, 2002), can be compared in the future, the selection of MNE and dSPM was motivated by their popularity in the MEG community and by the availability of their implementation in commonly used MEG toolboxes (such as FieldTrip). Likewise, we selected DICS as a representative method for the beamforming approach. While the DICS and LCMV methods should provide similar results in investigating narrow band-limited power of source activity, DICS is considered to be preferable (Westner et al., 2022). The SAM beamformer was originally proposed to determine an optimal orientation at each location that corresponds to the largest power of the estimated source (Robinson and Vrba, 1999). Although SAM and DICS/LCMV use slightly different algorithmic approaches, they will provide similar results if an optimal orientation at each location is used to convert the DICS/LCMV beamformer from a vector form to a scaler (Westner et al., 2022).

The utility and accuracy of the sECD approach for language mapping using MEG were demonstrated in a series of studies by Papanicolaou and colleagues (Simos et al., 1998; Zouridakis et al., 1998; Papanicolaou et al., 1999; Breier et al., 2000). In a MEG study incorporating a relatively large number (*n* = 100) of patients with epilepsy, they found that LI based on the number of consecutive sECD sources in perisylvian brain areas had a high degree of concordance (87%) with the Wada test (as the clinical gold standard) for language laterality (Papanicolaou et al., 2004). They reported that the LI based on the MEG sECD approach (with respect to the Wada test) provided excellent sensitivity (98%), positive (91%) and negative (96%) predictive value, and a good specificity (83%). In addition to these studies, several other groups have successfully investigated the language laterality based on the MEG sECD approach and reported very high concordance with the Wada test (Merrifield et al., 2007; Doss et al., 2009; Ota et al., 2011; see Bowyer et al. (2020b) for a comprehensive review).

While the results of previous studies have demonstrated the capability and accuracy of the sECD approach for language mapping, its main limitation, namely its dependence on subjective judgments, has prevented its general adoption. To address the limitations and difficulties in implementing the method consistently and optimally across investigators and centers, we developed the AsECDa which has the following main advantages over the manual sECD approach. (1) The AsECDa is objective as it is based on predefined parameters and is not influenced by a user’s subjective impressions or judgement. (2) An optimal subset of sensors (separately for the left and right hemispheres) at each millisecond is selected by the automatic sECD algorithm as opposed to the manual approach where the selection is based on subjective judgments. (3) Typically, in the manual implementation of the sECD for language mapping, a single dipole is fit in each hemisphere at a given time point. The AsECDa will fit multiple dipoles (up to five) at a given time point if a single dipole is not enough to model the measured MEG field pattern. This extends the number of dipoles that can modeled simultaneously, given the difficulty in visually identifying multiple isofield maps, and can fit more than two dipoles at a given latency. (4) The AsECDa utilizes an atlas in MNI space for selection of dipoles in language-specific ROIs while the manual sECD approach relies on subjective visual judgement on whether a dipole is within the language areas (based on the locations of the dipoles in the patient’s structural MRI in the native space). (5) Modifying key parameters (e.g., selected language ROIs, time window, and the rejection criteria for fitted dipoles) in the AsECDa can be easily and quickly performed, which may allow for a more comprehensive clinical evaluation of the language map of individual patients.

We found that the AsECDa outperformed MNE and dSPM, and particularly the DICS beamformer, regarding test–retest reliability of the language LI. Our results revealed an excellent test–retest reliability (Cor = 0.80) for calculation of LI using the AsECDa while MNE/dSPM and DICS-ERD in the alpha and low beta bands provided a fair to good test–retest reliability. On the other hand, DICS-ERD in the high beta and gamma bands and DICS-ERD in all the frequency bands failed to provide any significant (*p* > 0.1) correlation between the LIs of the two MEG sessions. In agreement with our findings for the high test–retest reliability of the LI using the sECD method, a previous study in 21 patients with epilepsy (who underwent two consecutive sessions of MEG recordings during the same WRT utilized in the current study) reported a good TRT for the manual sECD approach by two independent expert raters [Cor_Rater1_ = 0.6 and Cor_Rater2_ = 0.69] (Lee et al., 2006). Another MEG study had investigated the test–retest reliability of the dSPM and LCMV beamformer for localization of source activity in 20 healthy participants (Ala-Salomaki et al., 2021) using a different language task (i.e., picture naming). In agreement with our DICS beamformer findings, their LCMV beamformer results for the object naming task showed non-significant or poor test–retest reliability in modulations of oscillatory activity in the language-specific areas while this activity was consistent in the posterior cortical regions that are not typically associated with language processing. Additionally, they reported that dSPM for the picture naming task provided more consistent results compared to the LCMV beamformer in localization of source activity in the language-specific areas, which is similar to our results. Furthermore, the test–retest reliability of dSPM for the picture naming task was reported to be mostly fair to good in the left perisylvian language regions.

In comparison with the other methods, the AsECDa’s results were more consistent with prior studies regarding the expected atypical language representation in the epilepsy population. The DICS beamformer was biased toward atypical language representation (LI ≈ 0). In particular, the DICS-ERD in the alpha and low beta bands (which provided the most reliable results compared to the DICS-ERD in the other bands and DICS-ERS in all the frequency bands) showed a biased LI where more than 68% of the sessions were assigned an atypical language lateralization (i.e., a right lateralization or a bilateral language representation). Compared to DICS, the bias toward atypical representation was reduced for dSPM and MNE, where 50 and 55% of sessions showed atypical language representation, respectively. Similar to our dSPM results, a previous MEG study investigated language laterality using dSPM in 45 patients with epilepsy, during the same task (i.e., WRT) utilized in the current study, and reported an atypical language lateralization in 28.9–62.2% of patients based on different selections of ROIs (Raghavan et al., 2017). These results for DICS, MNE, and dSPM are in conflict with previous studies showing atypical language representation in 20–30% of epilepsy patients (Gaillard et al., 2007; Moddel et al., 2009; Moser et al., 2011; Dijkstra and Ferrier, 2013). For example, (Moser et al., 2011) estimated rates of (a)typical language dominance in 296 patients with epilepsy based on Wada testing scores and reported that approximately 25% of patients had atypical language laterality. Another study reported a slightly larger atypical language dominance in 30 out of 102 (29%) patients with epilepsy (Gaillard et al., 2007). In our current study, the AsECDa had the least bias toward atypical language representation (only 38% of the sessions) compared to the other methods. As argued previously in Papanicolaou et al. (2004), a slightly higher rate of atypical language representation obtained by the sECD approach compared to the Wada test may indicate lower specificity of the sECD approach such that more active sources in the nondominant hemisphere are detected.

Potential improvement in the results of dSPM using different methods for calculation of language LI was investigated by Tanaka et al. (2013). Using MEG recordings in 35 patients with epilepsy during a visual semantic decision task, they used dSPM and calculated language LI based on the following two approaches: (I) dSPM amplitude method, which is based on the sum of amplitudes of sources within language ROIs (similar to the method used in our current study); and (II) dSPM counting method, which involves counting the number of dipoles within language ROIs after applying a threshold set to half of the maximum amplitude of sources across the ROIs. Their results show that the dSPM counting method outperformed the dSPM amplitude method regarding concordance with the Wada test (91.4% vs. 51.4%). In addition, the dSPM counting and the dSPM amplitude method identified approximately 17 and 50%, respectively, of patients with atypical language lateralization. We implemented the dSPM counting and the dSPM amplitude method for calculation of the language LI in the current study and found no meaningful difference between the two methods. The counting method provided slightly better results regarding atypical language representation (48% vs. 50%) but worse test–retest reliability (Cor = 0.58 [*p* < 0.0059] vs. Cor = 0.64 [*p* < 0.0017]).

Use of the Wada test has declined in recent decades (Baxendale et al., 2008; Gross et al., 2022), and this test was only obtained in three out of the 21 patients in our study. The results of the AsECDa test matched with those of the Wada test for two patients, both of whom showed left language lateralization. For the third patient, the AsECDa results indicated bilateral representation for language, and the Wada test showed left lateralization. Although the Wada test results showed left lateralization for language in all three patients, the MNE and DICS beamformer results in the alpha band did not reveal left lateralization in any patient, resulting in a 0% concordance with the Wada test. Furthermore, the dSPM and DICS beamformer results in the beta band showed left laterality in one patient and bilateral or right laterality in two patients, resulting in a 33% concordance with the Wada test. Moreover, the laterality estimates of the AsECDa in this study closely match those of previous studies where Wada comparisons were made (see Papanicolaou et al. (2004) for example), indicating that the AsECDa is likely to have a comparable degree of concordance with the Wada procedure. Our findings for the advantage of AsECDa over MNE/dSPM and DICS beamformer (regarding test–retest reliability and the expected atypical language lateralization) are based on a receptive language task (i.e., WRT), which has been routinely used in our clinical practice and was shown to provide a high concordance between MEG and the Wada test language laterality (Papanicolaou et al., 2004).

The AsECDa was implemented for planar gradiometers of the MEGIN/Elekta system. As mentioned in Section 2.5, the AsECDa relies on identification of the local maxima of the planar gradients of the magnetic field. For the MEGIN/Elekta system, we combined the magnetic fields of two planar gradiometer channels to generate a single positive value per sensor location using the “*ft_combineplanar*” function in the Fieldtrip toolbox. However, this method can be adapted for other MEG systems. For example, the planar gradient can be approximated using the MEG signals in axial gradiometer sensors, such as in the CTF MEG system, with the help of the “*ft_megplanar*” function in the Fieldtrip toolbox. Nonetheless, we want to emphasize that the parameters of the AsECDa algorithm, such as the sigma factor and minimum and maximum radius in Eq. (1), are optimized based on the planar gradiometers of the MEGIN/Elekta system and may need adjustment for other MEG systems.

## 5. Conclusion

In this study, we developed a completely objective and automatic sECD algorithm (AsECDa) for language mapping using MEG that has several advantages over the manual sECD approach. Results obtained using synthetic MEG data (based on single dipoles located at the centroid of the 246 ROIs of the Brainnetome atlas) revealed a good localization accuracy for the AsECDa in both superficial and deep sources, with an average localization error at a typical noise level (SNR = 5) of less than 2 mm. Our results based on MEG data collected in patients with epilepsy during a receptive language task (i.e., WRT) in two consecutive sessions showed that the AsECDa outperformed MNE, dSPM, and DICS beamformer regarding test–retest reliability of the LI. We also found that the results of the AsECDa were in greater agreement with previous studies on the prevalence of atypical language lateralization in epilepsy patients, as opposed to the other source localization methods. Considering these results, we believe that the AsECDa is a reliable method that can be easily and confidently implemented in clinical evaluations or in research investigations.

## Data availability statement

The datasets presented in this article are not readily available because of the ethical permission. The data that support the findings of this study are available from the corresponding author with permission of the University of Texas at Austin and Ascension Site Approval Committee. Requests to access the datasets should be directed to AB-F, babajani.a@ufl.edu.

## Ethics statement

The studies involving human participants were reviewed and approved by the Institutional Review Board (IRB) of the University of Texas at Austin and Ascension Site Approval Committee. Written informed consent from the participants’ legal guardian/next of kin was not required to participate in this study in accordance with the national legislation and the institutional requirements.

## Author contributions

AB-F: conceptualization, methodology, algorithm development, software, data processing, investigation, writing – original draft, and writing – review and editing. HP: methodology, algorithm development, software, and writing – review and editing. WS, CC, and DC: conceptualization and writing – review and editing. AP: conceptualization, methodology, algorithm development, and writing – critically review and editing. All authors contributed to the article and approved the submitted version.

## Funding

This work was supported by the Clarke’s Family Foundation.

## Conflict of interest

The authors declare that the research was conducted in the absence of any commercial or financial relationships that could be construed as a potential conflict of interest.

The reviewer IM declared a past co-authorship with the author DC to the handling editor.

## Publisher’s note

All claims expressed in this article are solely those of the authors and do not necessarily represent those of their affiliated organizations, or those of the publisher, the editors and the reviewers. Any product that may be evaluated in this article, or claim that may be made by its manufacturer, is not guaranteed or endorsed by the publisher.

## Figures and Tables

**Table:** 

AsECDa	Automatic sECD algorithm
CSD	Cross-spectral density
Cor	Correlation
DICS	Dynamic imaging of coherent sources
DPSS	Discrete prolate spheroidal sequences
dSPM	Dynamic statistical parametric mapping
EMFs	Evoked magnetic fields
ERD	Event-related desynchronization
ERS	Event-related synchronization
FFT	Fast Fourier transform
FOI	Frequency of interest
ISI	Interstimulus interval
HPI	Head position indicator
LCMV	Linearly constrained minimum variance
LI	Laterality index
LM	Language mapping
MEG	Magnetoencephalography
MNE	Minimum norm estimation
MNI	Montreal Neurological Institute
ROI	Regions of interest
SAM	Synthetic aperture magnetometry
SD	Standard deviation
sECD	Single equivalent current dipole
SNR	Signal-to-noise ratio
sLORETA	Standardized low-resolution brain electromagnetic tomography
TRR	Test–retest reliability
tSSS	Temporal extension of signal space separation
WRT	Word recognition task

## Footnotes

^1^https://www.fieldtriptoolbox.org/tutorial/automatic_artifact_rejection/
