# Supplementary material for: MEG language mapping using a novel automatic ECD algorithm in comparison with MNE, dSPM, and DICS beamformer
Source: Front Neurosci. 2023 Jun 2;17:1151885. doi: 10.3389/fnins.2023.1151885 (PMC10272516; doi:10.3389/fnins.2023.1151885)
Supplement: Supplementary file 1 [file Presentation_1.pdf]

Supplementary Materials

1. *Language task protocol*

Patients completed an auditory word recognition task (WRT) for receptive language mapping. Patients were instructed to listen to and memorize five target words (‘*little*’, ‘*please*’, ‘*drink*’, ‘*jump*’, and ‘*good*’) imediatletly prior to the beginning of the MEG scan. During the MEG recordings, the patients were instructed to lift their right index finger when they recognized one of the five target words. The stimuli comprised three blocks, each of which included 40 (non-repeating) distractor words and the five target words in a random order (**Figure S1**). The duration of the words was 587 ± 86 [mean ± SD] ms. Stimuli were presented with a randomly varied interstimulus interval (ISI) ranging between 2000 to 3000 ms (2500 ± 280 [mean ± SD] ms).

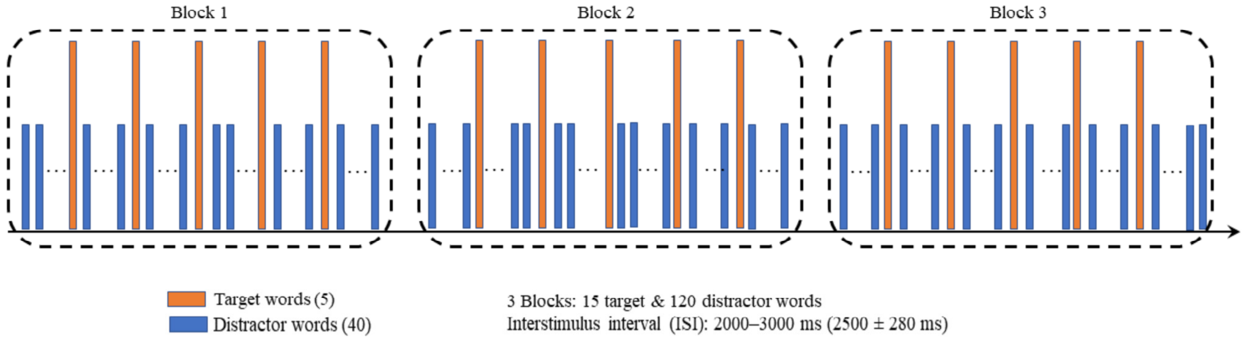

Figure S1. Stimuli design for the word recognition task used in this study.

2. *Language regions-of-interest*

We used language specific regions-of-interest (ROIs) in the Brainnetome atlas (Fan et al., 2016) for AsECDa and DICS beamformer (**Table S1-a**). For MNE and dSPM, the Destrieux standard atlas (Destrieux et al., 2010) was used for the language specific ROIs (**Table S1-b**).

Table S1. Language ROIs from (a) the Brainnetome volumetric atlas, which were used for automatic single equivalent current dipole algorithm (AsECDa) and dynamic imaging of coherent sources (DICS) beamformer, and (b) the Destrieux standard atlas, which were used for minimum norm estimation (MNE) and dynamic statistical parametric mapping (dSPM).

(a)

#	Language regions-of-interest (ROI)
1	IFG, Inferior Frontal Gyrus A44d, dorsal area 44
2	IFG, Inferior Frontal Gyrus IFS, inferior frontal sulcus
3	IFG, Inferior Frontal Gyrus A45c, caudal area 45
4	IFG, Inferior Frontal Gyrus A45r, rostral area 45
5	IFG, Inferior Frontal Gyrus A44op, opercular area 44

6	IFG, Inferior Frontal Gyrus A44v, ventral area 44
7	STG, Superior Temporal Gyrus A38m, medial area 38
8	STG, Superior Temporal Gyrus A41/42, area 41/42
9	STG, Superior Temporal Gyrus TE1.0 and TE1.2
10	STG, Superior Temporal Gyrus A22c, caudal area 22
11	STG, Superior Temporal Gyrus A38l, lateral area 38
12	STG, Superior Temporal Gyrus A22r, rostral area 22
13	MTG, Middle Temporal Gyrus A21c, caudal area 21
14	MTG, Middle Temporal Gyrus A21r, rostral area 21
15	MTG, Middle Temporal Gyrus A37dl, dorsolateral area37
16	MTG, Middle Temporal Gyrus aSTS, anterior superior temporal sulcus
17	ITG, Inferior Temporal Gyrus A20iv, intermediate ventral area 20
18	ITG, Inferior Temporal Gyrus A37elv, extreme lateroventral area37
19	ITG, Inferior Temporal Gyrus A20r, rostral area 20
20	ITG, Inferior Temporal Gyrus A20il, intermediate lateral area 20
21	ITG, Inferior Temporal Gyrus A37vl, ventrolateral area 37
22	ITG, Inferior Temporal Gyrus A20cl, caudolateral of area 20
23	ITG, Inferior Temporal Gyrus A20cv, caudoventral of area 20
24	FuG, Fusiform Gyrus A20rv, rostroventral area 20
25	FuG, Fusiform Gyrus A37mv, medioventral area37
26	FuG, Fusiform Gyrus A37lv, lateroventral area37
27	PhG, Parahippocampal Gyrus A35/36r, rostral area 35/36
28	PhG, Parahippocampal Gyrus A35/36c, caudal area 35/36
29	PhG, Parahippocampal Gyrus TL, area TL (lateral PPHC, posterior parahippocampal gyrus)
30	PhG, Parahippocampal Gyrus A28/34, area 28/34 (EC, entorhinal cortex)
31	PhG, Parahippocampal Gyrus TI, area TI (temporal agranular insular cortex)
32	PhG, Parahippocampal Gyrus TH, area TH (medial PPHC)
33	pSTS, posterior Superior Temporal Sulcus rpSTS, rostroposterior superior temporal sulcus
34	pSTS, posterior Superior Temporal Sulcus cpSTS, caudoposterior superior temporal sulcus
35	IPL, Inferior Parietal Lobule A39c, caudal area 39 (PGp)
36	IPL, Inferior Parietal Lobule A39rd, rostrodorsal area 39 (Hip3)
37	IPL, Inferior Parietal Lobule A40rd, rostrodorsal area 40 (PFt)
38	IPL, Inferior Parietal Lobule A40c, caudal area 40 (PFm)
39	IPL, Inferior Parietal Lobule A39rv, rostroventral area 39 (PGa)
40	IPL, Inferior Parietal Lobule A40rv, rostroventral area 40 (PFop)
41	INS, Insular Gyrus G, hypergranular insula
42	INS, Insular Gyrus vIa, ventral agranular insula
43	INS, Insular Gyrus dIa, dorsal agranular insula
44	INS, Insular Gyrus vId/vIg, ventral dysgranular and granular insula
45	INS, Insular Gyrus dIg, dorsal granular insula
46	INS, Insular Gyrus dId, dorsal dysgranular insula
47	Amyg, Amygdala mAmyg, medial amygdala
48	Amyg, Amygdala lAmyg, lateral amygdala
49	Hipp, Hippocampus rHipp, rostral hippocampus
50	Hipp, Hippocampus cHipp, caudal hippocampus

(b)

#	Language regions-of-interest (ROI)
1	G_Ins_lg_and_S_cent_ins
2	G_and_S_subcentral
3	G_front_inf-Opercular
4	G_front_inf-Orbital
5	G_front_inf-Triangul
6	G_insular_short
7	G_oc-temp_med-Parahip
8	G_pariet_inf-Angular
9	G_pariet_inf-Supramar
10	G_temp_sup-G_T_transv
11	G_temp_sup-Lateral
12	G_temp_sup-Plan_polar
13	G_temp_sup-Plan_tempo
14	G_temporal_inf
15	G_temporal_middle
16	at_Fis-post
17	Pole_temporal
18	S_circular_insula_ant
19	S_circular_insula_inf
20	S_circular_insula_sup
21	S_precentral-inf-part
22	S_temporal_inf
23	S_temporal_sup
24	S_temporal_transverse
25	S_interm_prim-Jensen

3. Effect of bandpass filters on MEG evoked magnetic fields (EMFs)

We used a 0.1-170 Hz bandpass filter for the DICS beamformer and 0.1-20 Hz bandpass filter for the other three methods (i.e., ECD, MNE, and dSPM). As shown in **Figure S2**, using a 0.1-170 Hz bandpass filter will not significantly change the time course of the average EMFs in the MEG sensors. Applying a 0.1-20 Hz bandpass filter instead of a 0.1-170 Hz bandpass filter on the MEG signals will reduce high frequency noise in the time course of the average EMFs. It is important to note that a 0.1-20 Hz bandpass filter cannot be used for the DICS beamformer if we wish to measure the power of brain signals in high beta (20-30 Hz), low gamma (30-50 Hz), and high gamma (50-110 Hz) bands.

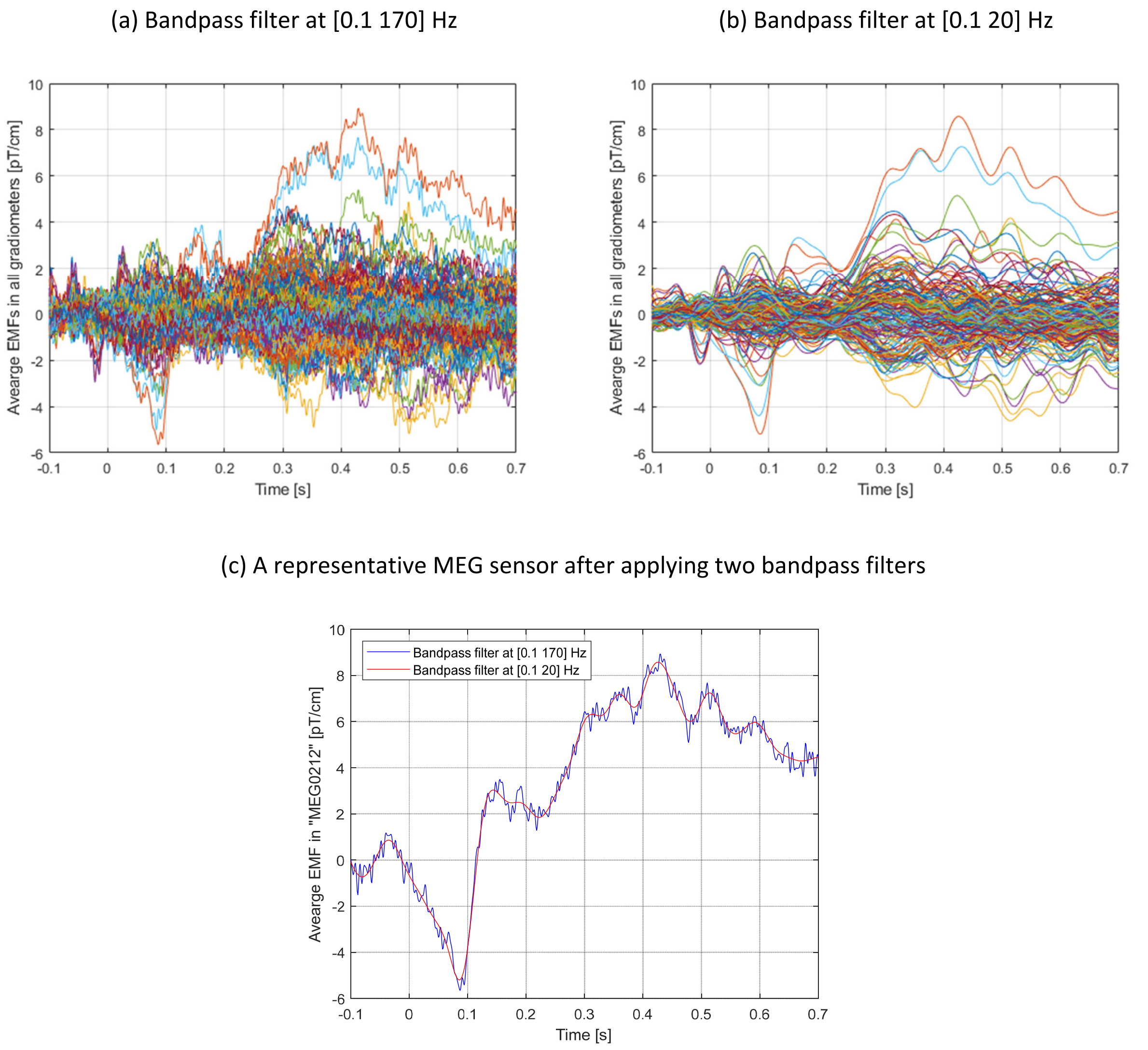

Figure S2. Effects of bandpass filters on the magnetoencephalography (MEG) evoked magnetic fields (EMFs). The top-left (a) and top-right (b) panels show the average EMF time courses of all gradiometers during the word recognition task in a representative patient using 0.1-170 Hz and 0.1-20 Hz bandpass filters, respectively. The bottom (c) panel shows the EMF signals of a representative MEG gradiometer (i.e., 'MEG0212') after applying 0.1-1700 Hz (blue color line) and 0.1-20 Hz (red color line) bandpass filters. Note that applying a 0.1-20 Hz bandpass filter instead of a 0.1-170 Hz bandpass filter reduced the high frequency noise in the time course of the average EMFs.

4. Impact of tSSS and Regularization on DICS Beamformer Results

The tSSS pre-processing reduces the rank of the cross-spectral density (CSD) of the MEG sensors, which is defined as Q(f) in Equation (9) of the manuscript, from 206 to approximately 70 (see **Figure S3**). To invert Q(f) in DICS (as described in Equation (8)), we handled the rank deficiency of CSD after tSSS by using a regularization parameter in the Fieldtrip toolbox, as "cfg.dics.lambda = '10%'". With this regularization parameter, we calculated the inversion of Q(f)

using the following formula in MATLAB: "invQf = pinv(Qf + lambda * eye(size(Qf)))", where lambda is calculated as 10% of the average of the eigenvalues of Q(f).

We performed experiments to evaluate the effect of different values of lambda on the DICS beamformer results, and found that using various values did not significantly improve the results. We also applied the DICS beamformer on MEG data without using the tSSS filter, and the results did not improve either.

To further investigate these findings, we reanalyzed MEG data of Pt# 18, who had a Wada test with a left lateralized language, using the DICS beamformer with different values of lambda and with/without tSSS filter. The results presented in **Table S2** demonstrate that different values of lambda did not significantly change the laterality index (LI) in MEG data with tSSS filter. Moreover, using MEG data without applying the tSSS filter reduced the consistency of the LI across different values of lambda, and generated an LI that showed a stronger tendency towards right lateralization, despite the fact that the subject was left lateralized based on the Wada test. We have included these results in the Supplementary Material.

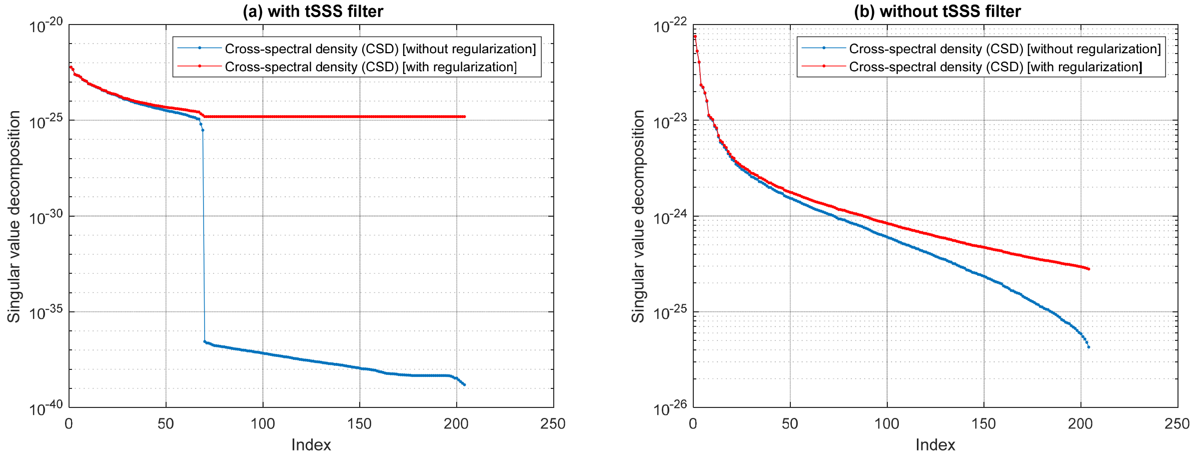

Figure S3. Singular value decomposition of the cross-spectral density (CSD) of the MEG sensors in the alpha band for a representative subject (Pt#18) in two pre-processing approaches: (a) with applying the tSSS filter and (b) without applying the tSSS filter. The blue line represents the CSD before applying regularization, while the red line represents the CSD after applying regularization at 10% of the average of the eigenvalues of the CSD.

Table S2. the results of the DICS beamformer in the alpha band with various lambda values, and the corresponding laterality index (LI) in Pt#18. The table illustrates that when MEG data was processed without the tSSS filter, the consistency of the LI across different lambda values decreased, and the LI tended to show stronger right lateralization, despite the fact that the subject had left lateralized language function according to the Wada test.

	lambda	Laterality Index	Laterality
without tSSS	0%	-0.12	Bilateral
	1%	-0.11	Bilateral
	5%	-0.13	Bilateral
	10%	-0.15	Bilateral
	15%	-0.20	Right
with tSSS	1%	-0.12	Bilateral
	5%	-0.09	Bilateral
	10%	-0.09	Bilateral
	15%	-0.08	Bilateral

REFERENCES

Destrieux C, Fischl B, Dale A, Halgren E. Automatic parcellation of human cortical gyri and sulci using standard anatomical nomenclature. Neuroimage 2010;53(1):1-15.

Fan L, Li H, Zhuo J, Zhang Y, Wang J, Chen L, et al. The Human Brainnetome Atlas: A New Brain Atlas Based on Connectional Architecture. Cereb Cortex 2016;26(8):3508-26.
